# Phosphatidylethanol levels and their association with withdrawal severity among individuals with severe alcohol use disorder seeking inpatient withdrawal management

**DOI:** 10.3389/fpsyt.2025.1567617

**Published:** 2025-06-13

**Authors:** Jeong Hoo Lee, Veronica Szpak, Lisa W. Vercollone, Peter R. Chai, Charlotte E. Goldfine, Samuel Maddams, Joji Suzuki

**Affiliations:** ^1^ Department of Psychiatry, Brigham and Women’s Faulkner Hospital, Boston, MA, United States; ^2^ Harvard Medical School, Boston, MA, United States; ^3^ Department of Psychiatry, Brigham and Women’s Hospital, Boston, MA, United States; ^4^ Department of Medicine, Dunedin Hospital, Southern Health New Zealand, Dunedin, New Zealand; ^5^ Department of Emergency Medicine, Brigham and Women’s Hospital, Boston, MA, United States

**Keywords:** alcohol use disorder, phosphatidylethanol (PEth), alcohol withdrawal, withdrawal severity, direct alcohol biomarker, brief research report

## Abstract

**Introduction:**

This study aimed to determine if phosphatidylethanol (PEth) levels are associated with alcohol withdrawal severity among individuals seeking inpatient withdrawal management.

**Methods:**

A prospective study enrolled individuals undergoing alcohol withdrawal treatment at a ASAM level 4 inpatient unit. Primary outcome was the association between serum PEth levels with alcohol withdrawal medication requirements in diazepam equivalent (mg). Secondary objectives examined associations between PEth levels, Clinical Institute Withdrawal Assessment for Alcohol (CIWA) scores, drinking history, Alcohol Use Disorders Identification Test (AUDIT), and Prediction of Alcohol Withdrawal Severity Scale (PAWSS) scores.

**Results:**

Thirty participants (mean age 48.7 years, SD 11.7; 67.7% white) reported average daily alcohol consumption of 14.2 drinks (SD 11.4, range 1.2-47.6) and percent heavy drinking days of 72% (SD 31.9, range 13.3-100). Nine (29%) reported history of withdrawal seizures and seven (22.6%) reported history of delirium tremens. Admission PEth (PO, ng/mL) levels (mean 934.9, SD 546.6; range 42 - 2000-) did not significantly associate with total medication requirements (r=0.05, p=0.78) or CIWA scores (r=0.09 to -0.14, p>0.05). PEth levels showed no significant correlations with AUDIT (r=0.17, p=0.35) or PAWSS scores (r=0.13, p=0.50). However, significant correlations were found between PEth levels and average drinks per day (r=0.54, p=0.002), as well as with the percentage of heavy drinking days (r=0.54, p=0.002).

**Discussion and conclusions:**

Consistent with prior reports, PEth levels appear to correlate with patients’ alcohol consumption including heavy drinking, but our results did not find that PEth levels predict alcohol withdrawal severity among heavy drinkers seeking inpatient withdrawal management. Further research is warranted to better understand the utility of PEth testing.

## 1 Introduction

Inpatient withdrawal management is often the first step in treatment for individuals with alcohol use disorder (AUD). Despite various methods, predicting withdrawal severity remains challenging. Phosphatidylethanol (PEth), a membrane-bound phospholipid, is synthesized exclusively in the presence of alcohol consumption (1, 2). Uniquely, studies have demonstrated PEth levels to correlate well with self-reported heavy drinking in the prior several weeks or more, making PEth a useful biomarker to assess the degree of recent drinking (3–6). Conversely, a negative PEth test indicates no heavy drinking in the past 1–2 weeks. Given these correlations, PEth levels may help assess withdrawal severity and guide treatment strategies. Currently, PEth testing is not commonly available in most clinical laboratories and samples often need to be sent to specialized reference laboratories for analysis (7–9). The processing time for PEth testing also typically ranges from 2 to 5 business days (7, 9). If baseline PEth levels reliably indicate withdrawal severity and guide treatment, it could encourage faster testing development.

A recent study found PEth levels to correlate with alcohol withdrawal symptoms in individuals with AUD seeking inpatient withdrawal management (10). To explore this relationship further, we conducted a prospective study with individuals undergoing inpatient alcohol withdrawal management.

## 2 Methods

Setting: We enrolled English-speaking adults aged 18 and older undergoing alcohol withdrawal treatment at an American Society for Addiction Medicine (ASAM) level 4 inpatient unit in an urban academic hospital. Level 4 units only admit patients at the highest risk for severe alcohol withdrawal, providing 24-hour intensive medical care, cardiac monitoring, pharmacotherapy, and intensive psychosocial support. Given this unique patient population, a fixed-dose regimen is universally utilized in which benzodiazepine or phenobarbital are administered proactively without waiting for escalating withdrawal scores. Clinical Institute Withdrawal Assessment (CIWA) scores are still obtained, but only to determine if additional pharmacotherapy is needed. The choice of medication and dose are determined by the individual attending physician, therefore introducing inter-individual variability in how withdrawal is managed. The study was approved by the Mass General Brigham Institutional Review Board (protocol#: 2023P000710) on 4/7/23 and recruitment took place from June to November of 2023.

Participants: Inclusion criteria were admission to the unit for alcohol withdrawal as assessed and determined by the addiction medicine team, English-speaking, and ability to provide informed consent. Exclusion criteria included impaired mental status (i.e. acute intoxication or delirium) that prevented informed consent and primary withdrawal management for substances other than alcohol based on the primary addiction team’s screening and urine drug test that was obtained on admission. Participants received compensation of $20 in the form of Forte card.

Study Procedures: Upon obtaining consent, we conducted the following assessments: Prediction of Alcohol Withdrawal Severity Scale (PAWSS) (11), Alcohol Use Disorders Identification Test (AUDIT) (12), and Time-Line Follow Back (TLFB) (13). TLFB was used to calculate drinks per drinking days and percent heavy drinking days in the prior 30 days. Blood sample was collected via standard phlebotomy procedure and the sample was then sent to the laboratory and analyzed using liquid chromatography-tandem mass spectrometry (LC-MS/MS) to detect and quantify PEth levels. The laboratory provided results for PEth homologues PO (16:0/18:1) PEth and PL (16:0/18:2) Peth. For our study, we utilized only PO PEth given that it is the most common (>75%) Peth homologue. Clinicians were not made aware of the PEth levels as they were obtained only for research purposes. Participants’ electronic medical records provided demographics, psychiatric and substance use history, laboratory tests, maximal CIWA scores on day 2 or 3 of admission assessed by nursing, and medications used in diazepam equivalents (10 mg diazepam = 2 mg lorazepam = 25 mg chlordiazepoxide = 30 mg IV phenobarbital) (14). Participants were continued on their home medications and did not receive additional psychiatric medications.

Outcomes: Primary outcome was medication requirement in diazepam equivalents.

Sample size calculation: Based on the prior study (10) that showed moderate effect size (0.5), we conducted an alph*a priori* power analysis to estimate that we would require a sample of 30 to achieve 80% power with alpha set at 5% to detect a significant association between PEth and medication requirement.

Analytic strategy: Analyses were conducted in R Studio version 4.2.2 (15). Descriptive statistics summarized the data. Pearson’s correlations were computed with the ‘cor.test ()’ function. Primary outcome assessed the correlation between PEth and medication requirements. Secondary outcomes explored the relationship between PEth levels with CIWA scores, drinking history, AUDIT, and PAWSS scores. Alpha was set at 0.05 for all analyses. In *post-hoc* analyses, different benzodiazepine conversions were examined as sensitivity analysis (15 mg diazepam = 2 mg lorazepam = 25 mg chlordiazepoxide = 30 mg IV phenobarbital) (14).

## 3 Results

The characteristics of those included are summarized in **Table 1**. Thirty participants were an average 48.7 years old (SD 11.7), and predominantly non-Hispanic white (67.7%) males (77.4%). Most were single (67.7%), employed (77.4%), and housed (77.4%). Common comorbid psychiatric conditions included depression (41.9%) and generalized anxiety disorder (51.6%). Substance use was prevalent, with tobacco (58.1%), cannabis (25.8%), cocaine (19.4%), opioids (12.9%), and benzodiazepines (9.7%). In the 30 days prior to admission, participants reported drinking on average 14.2 drinks per day (SD 11.4, range 1.2-47.6) with 72% heavy drinking days (range 13.3-100). Total medication requirement was on average 338.5mg (SD 210.6, range 57.5-860) diazepam-equivalent, maximal CIWA scores were on average 7.5 (SD 3.7, range 1-18), and PAWSS scores were on average 5.3 (SD 1.4, range 1-8). The average length of hospitalization was 4.7 days (SD 2.4, range 2-13).

**Table 1:** Participants Background Information.

Patient Characteristics	*n* = 31
Age, M (SD)	48.7 (11.7)
Sex, n, %	
Males	24 (77.4%)
Females	7 (22.6%)
Race, n, %	
White	21 (67.7%)
Black	8 (25.8%)
Other	2 (6.5%)
Ethnicity, n, %	
Hispanic	1(3.2%)
Non-Hispanic	30 (96.8%)
Marital Status, n, %	
Single	21 (67.7%)
Married/Life Partner	5 (16.1%)
Divorced	3 (9.7%)
Other	2 (6.5%)
Employment, n %	
Employed	24 (77.4%)
Unemployed	6 (19.4%)
Retired	1 (3.2%)
Home Status, n, %	
Domiciled	24 (77.4%)
Unstable Housing	7 (22.6%)
Psychiatric Diagnoses, n, %	
Major Depressive Disorder	13 (41.9%)
Bipolar Disorder	5 (16.1%)
ADD or ADHD	1 (3.2%)
Panic Disorder	2 (6.5%)
Post Traumatic Stress Disorder	4 (12.9%)
Generalized Anxiety Disorder	16 (51.6%)
Alcohol use disorder history	
Drinks per Day	14.2(11.4, 1.2-47.6)
Percent Heavy Drinking Days	72 (31.9, 13.3-100)
Withdrawal Seizure, n (%)	9 (29.0%)
Delirium Tremens, n (%)	7 (22.6%)
AUDIT, M, SD, Range	27.5 (7.3, 10-38)
PAWSS, M, SD, Range	5.3 (1.4, 1-8)
Substance use disorder history, n, %	
Opioids	4 (12.9%)
Cocaine	6 (19.4%)
Benzodiazepines	3(9.7%)
Cannabis	8 (25.8%)
Tobacco	18 (58.1%)
Admission, M, (SD, Range)	
Length of Stay (Days)	4.7 (2.4, 2-13)
Blood Alcohol Level (mg/dL)	182.0 (138.6, 0-495)
Maximal CIWA Score	7.5(3.7, 1-18)
PEth Level (PO, ng/mL)	934.9 (546.6, 42-2,000)
Medication requirement in diazepam equivalent (mg)	338.5 (210.6, 57.5-860)

**Figure 1:**
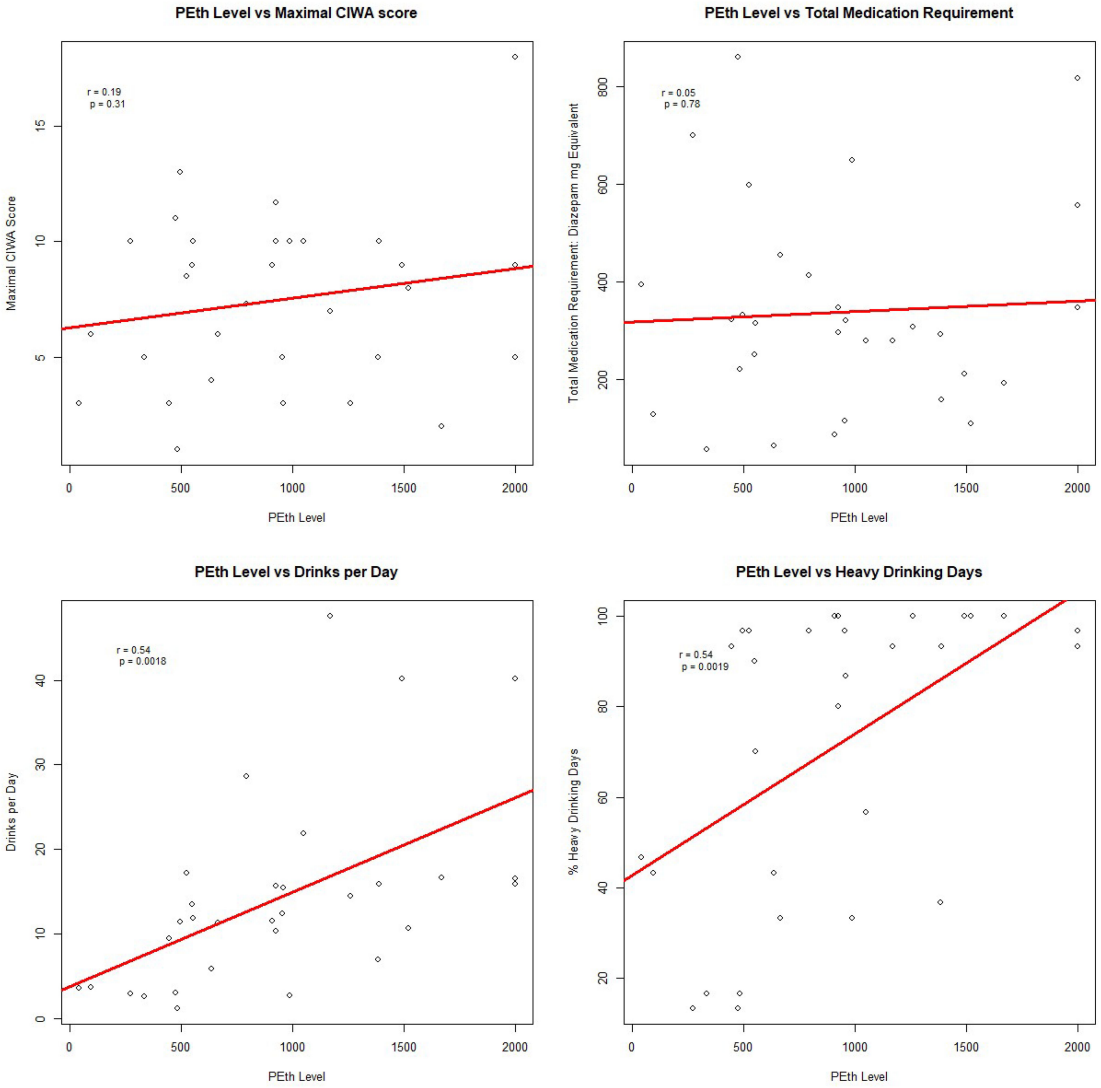
Pearson's Correlation Analysis.

Pearson’s correlation analysis (**Figure 1**) showed no significant correlation between PEth and medication requirements in diazepam equivalence (r=0.05, p=0.78; **Figure 1**). No significant correlations were found between PEth levels and CIWA scores (r=0.09 to -0.14, p>0.05), AUDIT scores (r=0.17, p=0.35), AUDIT-C scores (r=0.14, p=0.45), or PAWSS scores (r=0.13, p=0.50). There was a significant correlation between PEth levels and drinks per day (r=0.54, p=0.0018; **Figure 1**) and percent heavy drinking days (r=0.54, p=0.0019; **Figure 1**). *Post-hoc* analyses with different benzodiazepine conversions did not affect these results.

## 4 Discussion

We aimed to assess whether PEth levels could predict the severity of alcohol withdrawal because of recent positive study which found such a positive correlation (10). We therefore hypothesized a similar correlation, but our results tentatively led us to reject this hypothesis. The participants in our study generally presented with more severe drinking patterns compared to those in the previous study and were at higher risk for severe withdrawal. Our results therefore could potentially suggest a ceiling effect, where PEth’s utility in predicting withdrawal severity is limited beyond a certain threshold of alcohol use. Our study also used a fixed-dose regimen to treat alcohol withdrawal, as opposed to a symptoms-triggered approach, proactively managing withdrawal symptoms rather than waiting for escalating CIWA scores and ensuring that patients, even those at the highest risk, do not experience significant symptoms. The PEth testing for study had an upper limit 2000 ng/mL, which introduced limitations on assessing the exact correlation with PEth values higher than 2000 ng/ml. Taken together, while our study failed to demonstrate a correlation between PEth and withdrawal severity, more research is needed to determine if certain populations of AUD or settings may be better suited for PEth testing as a risk stratification tool to predict alcohol withdrawal severity. Currently, there is also a limitation with the delay in obtaining PEth levels, and further study findings regarding the new utility of PEth may necessitate and encourage the development of faster testing.

Consistent with the prior research, our results confirm that PEth levels correlate with recent self-reported alcohol consumption. Direct alcohol markers such as blood alcohol level and ethylglucuronide have narrow windows of detection limiting their utility in assessing recent drinking (16). Indirect biomarkers such as liver function tests or carbohydrate deficient transferrin also suffer from poor predictive value in identifying recent drinking (17). Our study therefore supports the use of PEth in addition to self-report as a valuable biomarker to ascertain the degree of recent drinking.

Our study has limitations. The study was performed in a single institution, on an ASAM level 4 inpatient unit, with a small sample size (and lack of diversity in ethnic/cultural, racial, socioeconomic background), limiting our ability to generalize to other settings or populations. While there were standardized approaches to managing withdrawal, each physician attending on the unit had considerable flexibility in the choice of medication and dose to use (42% of patients received phenobarbital or phenobarbital/benzodiazepine combination and 58% received benzodiazepines alone in this study), potentially introducing a confound which we did not control for in our analyses. Our results may not generalize to settings that predominantly utilize symptom-triggered approach, including ASAM level 3 withdrawal management units. Finally, our sample size estimates may have been inaccurate since we used the prior study by Novak et al. to estimate the effect size.

PEth level may not predict the severity of alcohol withdrawal for individuals with severe AUD at risk for severe withdrawal admitted to an ASAM level 4 inpatient withdrawal management unit. Further studies conducted in various settings, with a wider spectrum of AUD severity, and with adequate sample sizes are needed to better assess the possible utility of PEth as a predictor of alcohol withdrawal severity.

## Data Availability

The raw data supporting the conclusions of this article will be made available by the authors, without undue reservation.
